# Comprehensive Approaches for the Search and Characterization of Staphylococcins

**DOI:** 10.3390/microorganisms11051329

**Published:** 2023-05-18

**Authors:** Rosa Fernández-Fernández, Carmen Lozano, Rine Christopher Reuben, Laura Ruiz-Ripa, Myriam Zarazaga, Carmen Torres

**Affiliations:** Area of Biochemistry and Molecular Biology, OneHealth-UR Research Group, University of La Rioja, 26006 Logroño, Spain

**Keywords:** *Staphylococcus*, staphylococcin, applications, antimicrobial-resistance alternatives

## Abstract

Novel and sustainable approaches are required to curb the increasing threat of antimicrobial resistance (AMR). Within the last decades, antimicrobial peptides, especially bacteriocins, have received increased attention and are being explored as suitable alternatives to antibiotics. Bacteriocins are ribosomally synthesized antimicrobial peptides produced by bacteria as a self-preservation method against competitors. Bacteriocins produced by *Staphylococcus*, also referred to as staphylococcins, have steadily shown great antimicrobial potential and are currently being considered promising candidates to mitigate the AMR menace. Moreover, several bacteriocin-producing *Staphylococcus* isolates of different species, especially coagulase-negative staphylococci (CoNS), have been described and are being targeted as a good alternative. This revision aims to help researchers in the search and characterization of staphylococcins, so we provide an up-to-date list of bacteriocin produced by *Staphylococcus*. Moreover, a universal nucleotide and amino acid-based phylogeny system of the well-characterized staphylococcins is proposed that could be of interest in the classification and search for these promising antimicrobials. Finally, we discuss the state of art of the staphylococcin applications and an overview of the emerging concerns.

## 1. Introduction

Antimicrobial resistance (AMR) is considered one of the most relevant threats affecting not only human and animal health but also environmental health and food security. Unless AMR’s spread and associated infections are globally prioritized and mitigated, health and economic burdens across the world will continue to worsen. Therefore, the sustainable prevention of human and animal infections and the reduction of the transmission of foodborne and zoonotic pathogens is necessary for ensuring food safety and public health. The frequent detection of top-priority antibiotic-resistant pathogens, especially methicillin-resistant *Staphylococcus aureus* (MRSA), vancomycin-resistant *Enterococcus faecium*, carbapenem-resistant and extended-spectrum beta-lactamase (ESBL)-producing *Enterobacteriaceae*, carbapenem-resistant *Acinetobacter baumannii* and *Pseudomonas aeruginosa*, and fluoroquinolone-resistant *Campylobacter* spp. and *Salmonella* [1] within the human, animal, and environment/food systems pose a significant threat which could be exacerbated if urgent measures are not explored. In this respect, it has been reported that infections caused by MRSA are more difficult to treat and costlier for healthcare systems [2].

Within the last decades, several emerging therapeutic alternatives, including antimicrobial peptides, bacteriophages, nanomedicines, probiotics, phytochemicals, photodynamic light therapy, etc., are being explored as suitable alternatives to antibiotics [3,4,5,6]. Bacteriocins are antimicrobial peptides produced by bacteria, mainly of ribosomal synthesis [7] that have recently attracted immense interest due to their high antimicrobial activities and stability [8]. Bacteriocin production seems to be a common characteristic among microorganisms, and it has been reported that most bacteria synthesize at least one antimicrobial compound [9]. Moreover, bacteriocin production is considered a self-preservation mechanism that allows bacteria to outcompete other members of the community, interfere in communication with the host and/or other bacteria, and prevent colonization, setting up very robust communities [10,11,12].

*Staphylococcus* is a Gram-positive commensal bacterial genus of humans and animals and can also be found in diverse environments and food [13,14,15,16]. Some staphylococcal species, including coagulase-positive (CoPS) and coagulase-negative staphylococci (CoNS), have been described as bacteriocin producers, commonly termed as staphylococcins [17,18,19]. Interestingly, CoNS species are frequently found as commensals of humans and animals, being infrequently associated with infections. These characteristics make them excellent candidates for the research and development of safe and economical antimicrobial substances against antibiotic-resistant pathogens.

In recent times, increasing research focusing on the characterization, mechanisms, activity (mostly against *S. aureus*), safety evaluation (including cytotoxicity), and regulations of bacteriocins detected in staphylococci have been reported [7,19]. Recently, we identified and characterized some relevant bacteriocin-producing staphylococci from animal, human, and environmental sources in our laboratory [20,21,22].

In this review, a comprehensive and technical dossier for the search for and characterization of bacteriocins detected in staphylococci is presented as an interesting source of novel natural antimicrobial compounds with relevant interest to deal with the AMR problem. Furthermore, an extensively up-to-date classification of staphylococcal bacteriocins is provided, as well as a novel phylogeny classification of staphylococcin structural genes and proteins. Finally, a brief overview of possible bacteriocin applications is included.

## 2. Classification and Niche of *Staphylococcus*

Many staphylococcal species, both CoPS and CoNS, are found in humans, animals, and food [23]. They normally interact as commensal bacteria, although they can also act as opportunistic pathogens [24], especially CoPS, with *S. aureus* and *S. pseudintermedius* as the most reported cause of infections [25]. *S. aureus* is commonly related to skin and soft tissue infections in humans and cow mastitis [26]. As for companion animals, *S. pseudintermedius* is frequently isolated from pyoderma and postoperative dermatological infection cases in dogs [27]. Staphylococci are also common contaminants of animal-derived foods, such as raw meats or milk-derived products, and are responsible for most food toxi-infections in humans [23,24].

Staphylococci inhabit a wide diversity of polymicrobial environments and often compete for resources. Some commensal staphylococcal species can prevent the colonization of other pathogenic ones. Bacteriocin production is regarded as one of the defense mechanisms developed for self-preservation. Since bacteriocins, both CoPS and CoNS, are being considered as a new strategy to combat bacterial infections and the problem of antibiotic resistance, it is important to study their respective niche microbiota.

### 2.1. Staphylococci in Skin/Nasal Microbiota of Humans

Long-term bacterial residents isolated from the human skin microbiota include those from the genus *Staphylococcus*. It is estimated that 20–30% of the anterior nares are colonized by *S. aureus* [11,18], and the presence of this opportunistic pathogen has been linked to reduced bacterial diversity, exacerbated disease symptoms, and frequently precede infection [28,29,30]. This percentage of *S. aureus* carriers could be affected by risk factors such as occupational contact with farm animals [31].

Various CoNS species are known to inhabit the human skin microbiota, including *S. epidermidis*, *S. capitis*, *S. hominis*, *S. cohnii*, and *S. warneri*, among others [28,30]. *S. epidermidis* is known to be a core microbiome member of both the skin and nose, typically, while *S. warneri* has been found at a lower percentage than other CoNS but also in the nares and on the skin [30]. *S. lugdunensis* has been isolated from the human nose at an incidence rate of 10 to 26% [30]. Interestingly, some strains of these species have been found to negatively impact *S. aureus* viability, thus, preventing nasal *S. aureus* colonization or infections [11,30,32,33,34].

Over time, bacteriocin-producing staphylococcal isolates have been recovered from human skin and nasal microbiota. Among the well-described staphylococcins, we can highlight the Staphylococcin C55 [35,36], Bsa [37,38], Capidermicin [8], Endopeptidase ALE-1 [39], NisinJ [32,40], Nukacin IVK45 [41], Pep5 [42,43,44], Epidermicin NI01 [45], Epilancin 15X [46,47], Staphylococcin T (StT) [48], Hominicin [49,50], Lugdunin [51], and SWLP1 [19] bacteriocins. Moreover, the bacteriocins Epidermin [52,53,54,55,56] and Epicidin 280 [57] have been isolated from human clinical samples and an extensive number of bacteriocin-like-inhibitory-substances have also been detected in *Staphylococcus* of human origin, such as Staphylococcin BacR1 [58], Staphylococcin IYS2 [59], Staphylococcin Au-26 [60], Bac 201 [61], Staphylococcin 188 [62], Staphylococcin D91 [63], TE8 [64], and Hogocidinα/β [34] (Table 1 and Table 2).

Finally, several bacteriocins included in Table 1 and Table 2 are produced by isolates re-covered from environmental samples (Warnericin RK [65]) or laboratory strains (Staphylococcin 1580 [66], Bac 1829 [67], Lysostaphin [68,69] and Epilancin K7 [70]).

**Table 1:** Bacteriocins described in coagulase-positive staphylococci (CoPS) and coagulase-variable staphylococci (*S. hyicus*/*S. agnetis*).

Bacteriocin	Producer (Strain)	Origin	Activity against *		Classification ^a^	References
			Gram (+)	Gram (−)		
Staphylococcin C55	*S. aureus* (C55)	Human skin	*S. aureus*, streptococci, pneumococci, *Corynebacterium*, *Enterococcus*	*Neisseria*	Class II	[35,36]
Staphylococcin BacR1	*S. aureus* (UT0007) *S. aureus* (UT0002)	Clinical	*Staphylococcus*, *Streptococcus*, *Corynebacterium*, *Enterococcus*, *Bacillus*	*Neisseria*, *Haemophilus*, *Moraxella*, *Bordetella*, *Pasteurella*	BLIS	[58]
Aureocin A70	*S. aureus* (A70)	Milk	*Listeria monocytogenes*, *Staphylococcus*	−	Class II	[71,72]
Aureocin 4181	*S. aureus* (4181)	Bovine mastitis	*Staphylococcus*, *Streptococcus*	−	ClassII	[73]
Aureocin A53	*S. aureus* (A53)	Milk	Lactic acid bacteria, *L. monocytogenes*, *S. aureus*, *Mycobacterium bovis*	−	Class II	[74]
Aureocin 215FN	*S. aureus* (215FN)	Cow nare	*Corynebacterium*, *Streptococcus*, *L. monocytogenes*, *Bacillus*, *Lactobacillus*	−	BLIS	[75,76]
Staphylococcin 414	*S. aureus* (414)	Turkey	*Staphylococcus*, *Micrococcus*, *Bacillus*, *Lactobacillus*, *Streptococcus*	−	BLIS	[77]
Staphylococcin 462	*S. aureus* (462)	Mink	*S. aureus*	−	BLIS	[78]
Staphylococcin IYS2	*S. aureus* (IYS2)	Human saliva	*S. aureus*, *Streptococcus*, *Propionibacterium*, *L. monocytogenes*, *Corynebacterium*, *Actinomyces*	−	BLIS	[59]
Staphylococcin Au-26	*S. aureus* (26)	Human vagine	*Staphylococcus*, *Lactobacillus*, *Micrococcus*, *Streptococcus*	*Neisseria*	BLIS	[60]
Bac 1829	*S. aureus* (KSI1829)	Laboratory isolate *S. aureus* (RN4220)	*S. aureus*, *Streptococcus*, *Enterococcus*, *Corynebacterium*	*Haemophilus*, *Moraxella*, *Bordetella*, *Pasteurella*	BLIS	[67]
Bac 201	*S. aureus* (AB201)	Wound	*Staphylococcus*, *Streptococcus*, *Enterococcus*	*Neisseria*, *Acinetobacter*	BLIS	[61]
Staphylococcin 188	*S. aureus* (188)	Clinical	*Staphylococcus*, *Micrococcus*, *Streptococcus*, *Corynebacterium*, *Mycobacterium tuberculosis*	*Escherichia coli*, *Salmonella*, *Shigella*,	BLIS	[62]
Staphylococcin D91	*S. aureus* (D91)	Clinical	*Staphylococcus*, *Streptococcus*	*Proteus*, *E. coli*, *Pseudomonas*	BLIS	[63]
BacCH91	*S. aureus* (CH-91)	Poultry (DSM26258)	*Staphylococcus*, *Streptococcus*, *Micrococcus*	−	Class I	[79]
Bsa	*S. aureus* (MW2)	MRSA community-acquired (ST8, ST80)	*Staphylococcus*, *Micrococcus*	−	Class I	[37,38]
Aureocyclicin 4185	*S. aureus* (4185)	Bovine mastitis	*Listeria monocytogenes*, *Micrococcus*, *Bacillus*	−	Class IV	[80]
Hyicin/Agneticin 3682	*S. hycius/S. agnetis* (3682)	Bovine milk	*Staphylococcus*, *Listeria*, *Streptococcus*	−	Class I	[81]
Hyicin/Agneticin 4244	*S. hycius/S. agnetis* (4244)	Bovine mastitis	*Staphylococcus*, *Listeria* Anti-biofilm against *S. aureus*	−	Sactipeptide	[82]
BacSp222	*S. pseudintermedius* (222)	Dog skin	*Staphylococcus*, *Micrococcus*, *Streptococcus*, *Bacillus*	+	Class II	[83]

^a^ Staphylococcin classification in 6 groups (Class I–IV) also considering sactipeptides, thiopeptides, NRPs, and BLIS. * Antimicrobial activity: (+) positive; (−) negative.

**Table 2:** Bacteriocins described in coagulase-negative staphylococci (CoNS).

Bacteriocin	Producer (Strain)	Origin	Activity against *		Classification ^a^	References
			Gram (+)	Gram (−)		
Capidermicin	*S. capitis* (CIT060)	Human skin	*Bacillus*, *Enterococcus*, *Lactococcus*, *Micrococcus*, *Staphylococcus*	−	Class II	[8]
Endopeptidase ALE-1	*S. capitis* (EPk1)	Clinical sample	*+*	−	Class III	[39]
NisinJ	*S. capitis* (APC2923)	Human skin	*Listeria*, *Lactobacillus*, *Staphylococcus*, *Streptococcus*, *Corynebacterium*, *Enterococcus*	−	Class I	[32,40]
TE8	*S. capitis* (TE8)	Human skin	*S. aureus*	−	BLIS	[64]
Nukacin L217	*S. chromogenes* (L217)	Bovine teat apices	*Staphylococcus*, *Streptococcus*	−	Class I	[84]
Staphylococcin T (StT)	*S. cohnii* (T)	Healthy human	*Staphylococcus*, *Streptococcus*, *Micrococcus*, *Listeria*	*Neisseria*	Class I	[48]
NukacinIVK45	*S. epidermidis* (IVK45)	Human nasal	*Micrococcus*, *Corynebacterium*, *Streptococcus*, *Dolosigranulum pigrum*	−	Class I	[41]
Pep5	*S. epidermidis* (5)	Clinical	*Staphylococcus*, *Micrococcus*, *Corynebacterium*	−	Class I	[42,43,44]
Epicidin 280	*S. epidermidis* (BN 280)	Clinical	*Staphylococcus*	−	Class I	[57]
Epilancin K7	*S. epidermidis* (K7)	Laboratory strain	+	−	Class I	[70]
Epidermin	*S. epidermidis* (Tü 3298)	Clinical	+	−	Class I	[52,53,54,55,56]
Epidermicin NI01	*S. epidermidis* (224)	Clinical	*S. aureus*, *Enterococcus* Anti-biofilm against *S. epidermidis*	−	Class II	[45]
Epilancin 15X	*S. epidermidis* (15X154)	Clinical	*Staphylococcus*, *Enterococcus*	−	Class I	[46,47]
Staphylococcin 1580	*S. epidermidis* (1580)	Laboratory strain	*Staphylococcus*, *Streptococcus*, *Bacillus*, *Corynebacterium*, *Listeria*, *Acinetobacter*	−	BLIS	[66]
Micrococcin P1	*S. equorum* (WS 2733)	Cheese	*S. aureus*, *Enterococcus*, *Listeria*	−	Thiopeptide	[85]
Gallidermin	*S. gallinarum* F16/P57 Tü3928	Chicken	*Propionibacterium*, *Staphylococcus*, *Streptococcus*, *Micrococcus* Anti-biofilm against *S. aureus*	*Neisseria*, *Moraxella*	Class I	[86,87,88,89,90]
Hominicin	*S. hominis* (MBBL 2-9)	Healthy human	*S. aureus*, *Micrococcus*, *Bacillus*, *Lactobacillus*	*+*	Class I	[49,50]
Nukacin KQU-131	*S. hominis* (KQU-131)	Thai fermented fish Pla-ra	Lactic acid bacteria, *Micrococcus*, *Bacillus*	−	Class I	[91]
Hogocidin-α Hogocodin-β	*S. hominis* (A9)	Human skin	*S. aureus*	−	BLIS	[34]
Homicorcin	*S. hominis* (MBL_AB63)	Seeds	*Staphylococcus*, *Micrococcus luteus*, *Bacillus subtilis*, *Lactococcus lactis*		Class I	[92]
Lugdunin	*S. lugdunensis* (N920143)	Human nasal	*S. aureus*, *Enterococcus*	−	NRPs	[51]
Nukacin 3299 Simulancin 3299	*S. simulans* (3299) *S. simulans* (Ec105)	Bovine mastitis	*Staphylococcus*, *S. agalactiae*, *Corynebacterium*	−	Class I	[93]
Lysostaphin	*S. simulans biovar staphyIolyticus* (ATCC1362)	NRRL B-2628	*Staphylococcus*	−	Class III	[68,69]
Warnericin RB4	*S. warneri* (RB4)	Rice	*Thermo-acidophiles*, *Alicyclobacillus*, *Micrococcus*	−	Class I	[94]
Warnericin RK	*S. warneri* (RK)	Environmental	+	*Legionella*	BLIS	[65]
SWLP1	*S. warneri* (DSM 16081)	Human skin	+	−	Class I	[19]
Nukacin ISK-1	*S. warneri* (ISK-1)	Fermented rice bran “Nukadoko”	*Staphylococcus*, *Streptococcus*, *Micrococcus*, *Lactococcus*, *Bacillus*	−	Class I	[95,96,97,98,99]

^a^ Staphylococcin classification in 6 groups (Class I–IV) also considering sactipeptides, thiopeptides, NRPs, and BLIS. * Antimicrobial activity: (+) positive; (−) negative.

### 2.2. Staphylococci in Skin/Nasal Microbiota of Animals

Animal nasal and skin microbiota has been broadly analyzed, mostly for their carriage of pathogenic CoPS species such as *S. aureus* and *S. pseudintermedius*. On the other hand, several other studies also focused on multiple members of the animal skin/nasal microbiota, including CoNS.

Livestock often acts as a reservoir for livestock-associated MRSA (LA-MRSA), a variant of *S. aureus* with worldwide distribution among different animal species, which remains a serious public health threat [100]. The most relevant subgroup of LA-MRSA, the clonal complex (CC) 398, is known to colonize livestock, especially pigs, as well as humans in contact with pigs. A high prevalence of LA-MRSA CC398 is usually detected in regions with intensive pig farming [101,102]. *Staphylococcus* is very frequent in the skin and nasal microbiota of pigs, and *S. aureus is* commonly detected, together with other CoNS species such as *S. equorum*, *S. schlifieri*, *S. cohinii*, *S. chromogenes*, *S. haemolyticus*, *S. hyicus*, and *S. microti*, among others [103,104,105,106].

Moreover, nasal microbiota plays an important role in individual predisposition to *S. aureus* nasal carriage in pigs [107]. In this respect, studies evaluating the pig microbiota revealed that *S. aureus* colonization is also linked with the absence of *S. sciuri*, *S. cohnii*, or *S. saprophyticus* [108]. Although both *S. aureus* and *S. sciuri* have been found colonizing pigs [106], it seems that *S. sciuri* is more frequent in animals where *S. aureus* is less frequent in the nasal microbiota [109].

Recent studies have confirmed the high frequency of colonization of staphylococci in wild animals. CoPS species were commonly found in nasopharynx and rectal samples of free-ranging mammals recovered in Spain [110,111]. Moreover, CoNS isolates have been detected among 60–75% of wild birds, according to studies performed in Spain and Portugal [112,113], and in 38% of wild mammals [114].

Some mammal species, such as wild boars, mouflons, and deers, are frequently colonized by MSSA, *S. pseudintermedius*, and *S. hyicus* staphylococcal species [110]. *S. aureus*, especially the LA-MRSA CC398 genetic lineage, was the most frequent CoPS species detected [111]. Remarkably, hedgehogs and wild rabbits could be reservoirs of MRSA carrying the *mecC* gene, which could be a risk to human health [110,115,116,117].

According to CoNS, *S. sciuri* was the most common colonizer of healthy wild animals. With respect to wild birds, *S. lentus* was the second most frequently recovered species and has also been detected in farm animals and people with professional exposure to livestock [118,119,120]. *S. xylosus* and *S. chromogenes* were also frequently detected in wild boars [114].

Focusing on companion animals, *S. pseudintermedius* is a CoPS commonly found in the normal nasal and skin microbiota of dogs and is considered one of the most frequent bacterial pathogens isolated from clinical samples in these animals [27]. Moreover, it highlights the emergence of methicillin-resistant *S. pseudintermedius* (MRSP) [121] as a significant health problem [122]. Studies assessing the commensal staphylococci in pets revealed CoNS as the predominant (89%) microbial group of the bacterial community of the nasal cavity of healthy dogs [123,124].

On the other hand, a comparative analysis performed in 2019 by Gómez–Sanz and collaborators showed that although CoPS was predominant in owners and pets, MRCoNS, especially methicillin-resistant *S. epidermidis* (MRSE), are common colonizers of healthy owners and pets [125]. The co-carriage of CoPS and MRCoNS highlights the relevance of companion animals as reservoirs of important multidrug-resistant opportunistic pathogens, which can be transferred to in-contact individuals.

As for bacteriocin production, several staphylococcins have been reported among staphylococci of livestock (Table 1 and Table 2), frequently in those recovered from bovine mastitis (Aureocin 4181 [73], Aureocyclicin 4185 [80], Hyicin/Agneticin 4244 [82], Nukacin L217 [84], and Simulancin 3299 [93]). Moreover, several staphylococcins such as Aureocin 215FN [75,76], Staphylococcin 414 [77], Staphylococcin 462 [78], and BacCH91 [79] have also been detected in cow nares, turkey, mink, and poultry, respectively. On the other hand, the detection of bacteriocin-producing staphylococci in pets is very limited, and as far as we know, only two bacteriocins have been reported in isolates of dogs (BacSp222, *S. pseudintermedius*) and cats (Micrococcin P1, *S. felis*) [83,126]. There is little information about the detection of staphylococcins in staphylococci of wild animals. However, in a recent study, antimicrobial substances of *Staphylococcus* from migratory birds were detected and their potential role in nasotracheal microbiota modulation was analyzed [20].

### 2.3. Staphylococcus in Food

*Staphylococcus* isolates can be present in animal-derived food products. *S. aureus* has been related to food poisoning due to the production of enterotoxins, while CoNS species are not commonly involved in any case of staphylococcal infection [127,128]. However, CoNS can also carry enterotoxin genes, such as *S. saprophyticus* and *S. epidermidis* species, considered opportunistic pathogens [129].

A wide list of CoNS species has been well described in fermented meat, sausages, fermented fish, milk, cheese, and, more recently, in fermented soybean [16]. Moreover, *S. pasteuri* has been found in a large percentage (65.7%) of drinking water [23], and other species of the genus *Staphylococcus* have been reported in raw pork, chicken, and beef meat [130]. In recent years, *Staphylococcus* spp. have attracted the attention of worldwide researchers due to their relevant role in improving the organoleptic properties (texture, acidity, and flavor) of fermented food products [131]. One of the most remarked benefits of staphylococci in food is the stabilization capacity of the red color of meat products, derived through the production of nitrate reductase, an action that also inhibits foodborne pathogens [132].

Several CoNS species have typically been associated with fermented foods (sausages and meat-based items) and used as starter cultures [133]. Among them, *S. xylosus* and *S. carnosus* are the CoNS species most frequently applied as starter cultures to standardize production and inhibit foodborne pathogens [16].

Staphylococcin-producing isolates in food have been widely reported in the literature, especially in milk and fermented food (Hyicin 3682 [81], Aureocin A70 [71,72], Aureocin A53 [74], Gallidermin [86,87,88,89,90], Micrococcin P1 [85], Nukacin ISK-1 [95,96,97,98,99], Nukacin KQU-131 [91], Warnericin RB4 [94] and the recently discovered Homicorcin [92]) (Table 1 and Table 2). Moreover, recent works have reported the presence of bacteriocin-producing CoNS isolates in chicken-derived food that can act as protectors against other contaminant or pathogenic bacteria. The detection of two *S. sciuri* isolates with high antimicrobial activity is notable [21].

## 3. The Rationale for Exploring Bacteriocin-Producing *Staphylococci*: Beneficial and Functional Properties

The dearth of production of new and potent antimicrobial agents has led the scientific community to explore creative and unconventional remedies for AMR, including microorganisms and their products. Different studies have reported interest in bacteriocin-producing *Staphylococcus* isolates as noticeable sources of therapeutic agents.

Moreover, the ubiquitous nature of staphylococci, added to their flexible, multifaceted, and versatile metabolism, allow them to survive and inhabit highly diverse and distinct niches ranging from biotic and abiotic surfaces, environments, animals, humans, plants, (fermented) food, etc. Unlike other microbial groups, staphylococci are robust to environmental stresses, for example, acidic pH, the presence of the host’s antimicrobial peptides, regular UV radiation exposure, dryness, constant environmental changes, and perturbations, among others [134,135].

Strains from different staphylococcal species, such as *S. xylosus*, *S. simulans*, and *S. equorum*, can tolerate harsh environmental stresses, such as high concentrations of salts (up to 21%) thanks to the possession of membrane pumps, voltage-gated channels, and accumulation of glycine betaine [134,136,137]. Additionally, most staphylococci are often robust toward nitrogen metabolism and oxidative pressures [138].

On the other hand, recent studies have reported their lipolytic and proteolytic activities [139,140]. Some strains of staphylococci often degrade amino acid-derived biogenic amines [141], and depending on the species and strain, staphylococci disintegrate fatty acids, resulting in the formation of methyl ketones [142]. With respect to the metabolism of carbohydrates, several staphylococcal strains usually produce organic acids depending on oxygen availability [133,143]. In this respect, *Staphylococcus* is a suitable microbial group to be explored for techno-functional aspects, including bacteriocins and ecological interests [144].

## 4. Bacteriocins: Promising Antimicrobial Substances

Currently, many natural peptidic antimicrobials have been discovered, and they usually fall into one of these three classes: ribosomally-synthesized peptides (RSAPs), ribosomally-synthesized and post-translationally modified peptides (RiPPs) or peptides produced by non-ribosomal peptide synthetases (NRPs) [145]. Most of the bacteriocins are ribosomally-synthesized and have been generally described as small, heat-tolerant, broad-spectrum proteinaceous substances that may act on target cells in a variety of different mechanisms [146].

### 4.1. Staphylococcins: Classes and Diversities

*Staphylococcus* is a well-known bacteriocin-producing genus [17], and staphylococcins constitute a relatively narrow group of compounds. Staphylococcins are defined as antimicrobial peptides or proteins produced by staphylococci [19].

According to the classical bacteriocin classification, staphylococcins have been commonly divided into four groups of peptides and proteins (Class I–IV) [18,19]: (A) Class I bacteriocins, heat-stable and post-translationally modified small peptides known as lantibiotics (<5 kDa, 19–37 amino acids) [147]; (B) Class II bacteriocins, non modified post-translationally and heat-stable and small peptides (<10 kDa) [148]; (C) Class III bacteriocins, large (>30 kDa) and heat-labile peptides subdivided as lytic and non-lytic bacteriocins [149]; (D) Class IV bacteriocins, cyclic peptides formed by the post-translationally covalent linkage [150]. Moreover, other bacteriocins of the sactipeptides and thiopeptides groups have been recently discovered [151,152]. In this sense, recent reviews proposed to divide staphylococcins into six classes to better understand the characteristics of each group of antimicrobial peptides. These consider placing Aureocyclicin 4185 into Class IV, the first cyclic bacteriocin described in *Staphylococcus*; sactibiotics as Hyicin/Agneticin 4244 into Class V; and thiopeptides, as Micrococcin P1, into Class VI [19].

In addition to these bacteriocin classes, there are other types of NRP antimicrobials peptides with non-ribosomal synthesis (NRPs) [153] and among them, lugdunin pro-duced by *S. lugdunensis* is worth noting as an NRP produced by *S. lugdunensis* [51]. Moreover, there is another group of antimicrobial substances that act as bacteriocins but are neither obtained in pure form nor fully characterized. These substances known as Bacteriocin-Like Inhibitory Substances (BLIS) have been reported in the literature since 1991 [154]. Despite the unclear characteristics of BLIS, it should be noted that the unclear chemical structure of these compounds often does not limit even advanced applicative studies on these substances [155]. The presence of BLIS-producing staphylococcal isolates was recently reported among 60 of 890 staphylococcal isolates (6.7%) of different species and origins [21].

### 4.2. Biochemical and Genetic Characterization of Staphylococcins

To date, 47 staphylococcins have been fully identified and characterized depending on whether the producing *Staphylococcus* strain is considered CoPS or CoNS, respectively (Table 1 and Table 2).

Regarding CoPS, 20 staphylococcins have been reported, including those produced by *S. aureus* (aureocins of Class I, II, IV or BLIS), *S. pseudintermedius* (BacSp222, Class II), or the coagulase-variable staphylococci *S. agnetis* (Hyicins/Agneticins of Class I or Class V) (Table 1). Aureocins such as BacCH91 and Bsa, considered lantibiotics, are generally included in Class I and are mostly active against Gram-positive pathogens. Nevertheless, aureocins have also been described in Class II (Staphylococcin C55, Aureocin A70, the newly described variant Aureocin 4181 and Aureocin A53) and Class IV (Aureocyclicin 4185). Other aureocins without a completely determined gene or protein sequence are considered BLIS, and their characteristics are also shown in Table 1.

The 27 CoNS bacteriocins presented in Table 2 are mostly lantibiotics (Class I), but also bacteriocins of Classes II (Epidermicin NI01 and Capidermicin), III (Lysostaphin and Endopeptidase ALE-1), NRPs (Lugdunin), and 4 BLIS (TE8, Hogocidinα/β, Staphylococcin 1580, and Warnericin RK).

## 5. Bacteriocin Detection and Characterization Methods

To succeed in the detection of novel antimicrobial peptides, a rational selection of the environmental source of potential producers is a crucial step. Moreover, culture conditions and nutrient requirements should be carefully considered before the screening process. In this respect, it is known that changes in the natural environment of the producing isolate affect antimicrobial peptide synthesis, especially under in vitro conditions. In addition to the fact that bacteriocin production tends to occur against a narrow spectrum of bacteria, the detection and identification of the producing strains could be difficult. Here, we present a summary of the most common methodologies used to search for new antimicrobial compounds and suggestions for bacteriocin detection.

### 5.1. Phenotypic Methods

Multiple techniques have been used to identify and screen bacterial isolates for bacteriocin production in vitro. Frequently, agar diffusion assays are employed to evaluate the antimicrobial activity of potentially producing isolates using spot-on-lawn [156,157].

Moreover, diffusion assays are performed to evaluate the bioactivity of antimicrobial agents prior to and after the pre-purification process by comparing the zones of activity obtained with cell-free supernatants as well as the whole-cell extracts obtained after chemical extraction procedures against indicator bacteria [157]. In this respect, the extract of the producing strain is aseptically applied to blank discs (about 6 mm in diameter) or wells (diameter of 6 to 8 mm) and then introduced onto a plate previously seeded with the indicator microorganism (target). Multiple variations using specific culture media and various incubation conditions could be followed [158].

Moreover, other diffusion methods have been reported to screen the antimicrobial activity of extracts, fractions, or pure substances or to investigate the antagonism between microorganisms. Among these techniques, the agar plug diffusion and cross streak methods are the most commonly used [158].

However, all these methods have limitations because they cannot discriminate between inhibitory activity caused by bacteriocins or other antimicrobial substances [17]. Therefore, for a deeper physicochemical characterization of the antimicrobial agent’s nature, an overnight culture of the bacteria could be prepared, and later, a cell (or viable cell) free extract could be obtained and characterized. The effect of proteolytic enzymes, different temperatures and times of incubation, and several ranges of pH values are commonly evaluated [159,160]. Other studies have also tested the effect of organic solvents (alcohols, phenols) and salts [161].

### 5.2. Genotypic Methods

Genes encoding bacteriocins, as well as those genes encoding a set of immunity proteins and other accessory proteins, are arranged in operon clusters that reside in either chromosomes, plasmids, or other mobile genetic elements. The ribosomal synthesis and the presence of a self-defense immunity system distinguish bacteriocins from secondary metabolites that also exhibit antimicrobial activity [162]. Commonly, the detection of bacteriocin structural genes has been carried out through PCR and/or DNA/DNA hybridization [163,164].

The bacteriocin PCR matrix is based on known bacteriocin-related genes from the databases. To date, this method is actively used for screening bacteria that produce lantibiotics. However, due to each producing strain usually carrying different gene sequences or slight variations, PCR analysis and primer pairs should be optimized to avoid unspecificities [165].

Although a wide variety of bacteriocin genes have been described, there is no method based on PCR that allows the detection of several staphylococcins from a preliminary screening. Thankfully, since the advent of in-silico screening, this process of bacteriocin discovery has been significantly reduced in terms of time and cost. Moreover, the new genome mining tools offer an important technological resource in the discovery of novel natural products based either on the detection of bacteriocin structural genes or other bacteriocin-associated genes [166]. A wide variety of bioinformatic tools such as BACTIBASE, antiSMASH, BAGEL, APD3, ANTIMIC, DRAMP, or URMITE have been described [167,168,169,170,171]. However, it is noteworthy that harboring the staphylococcin gene clusters does not necessarily imply peptide production; thus, bacteriocin production should be confirmed by the antagonism assays explained above after finding those genes in the genomes [19].

### 5.3. Protein Methods

For bacteriocin detection and purification, it is important to verify the optimal conditions of production, and it is recommended to test the resulting eluents to verify their antimicrobial activity. Since bacteriocins form an extremely heterogeneous group of substances, specific purification protocols generally need to be designed. Three major bacteriocin purification methods can be distinguished according to the biochemical structure. (1) Subsequent ammonium sulfate precipitation, ion exchange, hydrophobic interaction, gel filtration, and reversed-phase high-pressure liquid chromatography [172], (2) a protocol based on simple three-step phases starting with ammonium sulfate precipitation, continuing with chloroform/methanol extraction/precipitation, and finishing with reversed-phase high-pressure liquid chromatography, as the sole chromatographic step involved [173], and (3) bacteriocin isolation through a unique unit operation. This last protocol can distinguish between the expanded bed adsorption method [174] and the use of organic solvents such as butanol [51].

After purification, MALDI-TOF mass spectrometry can be used for quick bacteriocin detection, and chromatograms should be examined for the identification of a known bacteriocin. Moreover, the presence of multiple peaks may indicate the presence of more than one peptide [157]. Thus, a combination of reverse-phase high-performance liquid chromatography (HPLC) and MALDI-TOF mass spectrometry can be used to determine if a purified substance obtained from the pooled active fractions contains a single, active bacteriocin or if multiple peptides are present [157].

In conjunction with peptide purification, genomic analysis for the identification of bacteriocin gene clusters is required to determine the novelty of the recovered antimicrobial peptide, and for the identification of new bacteriocins, ultra-HPLC coupled with mass spectrometry is recommended.

As a help for researchers, we present in this review a complete guide for staphylococcin identification. Table 3 shows a total of 27 structural bacteriocin nucleotidic and amino acid sequences registered on the NCBI databases until January 2023 and summarizes their associated genes, their accession GenBank number and GenePept sequences, their gene position, gene size (bp), and bacteriocin masses (Da). This information is of great use for staphylococcin detection both with PCR and whole genome analysis but also for their verification with mass spectrometry analysis.

### 5.4. Universal Nucleotide and Amino Acid-Based Staphylococcin Phylogenetics

**Figure 1:**
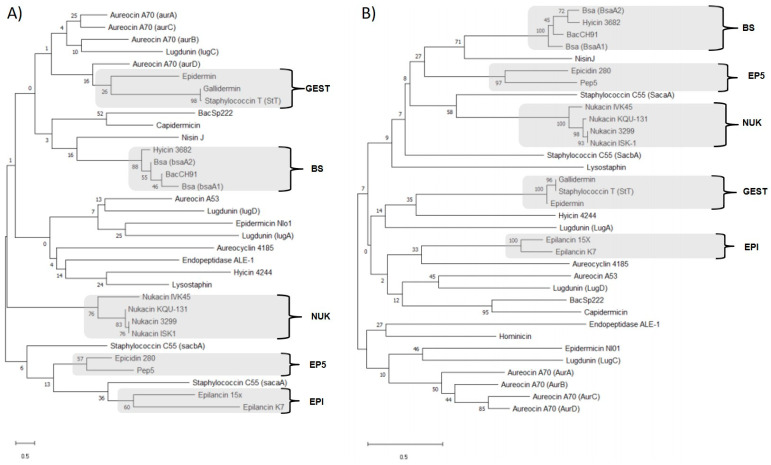
Bacteriocin similarities and staphylococcin families determined by the phylogenetic analysis carried out with the program MegaX: (**A**) Nucleotide-based phylogenetic tree and (**B**) Amino acid-based phylogenetic tree. In grey are marked the proposed bacteriocin families.

Here, we present a novel tool for bacteriocin detection in order to help researchers in their search and characterization. All structural genes and coding amino acids of well-described staphylococcins were selected (Table 3) (consult entry databases), and a phylogenetic analysis was conducted in MEGA X [175] based on the maximum likelihood homology of the sequences included (Figure 1). Thus, 34 amino acids and 33 gene sequences were used. The relationships were inferred using the neighbor-joining method [176]. The percentage of replicate trees in which the associated taxa clustered together in the bootstrap test (1000 replicates) is shown next to the branches [177]. The tree is drawn to scale 0.5, with branch lengths in the same units as those of the evolutionary distances used to infer the phylogenetic tree. The evolutionary distances were computed using the Poisson correction method [178]. All ambiguous positions were removed for each sequence pair (pairwise deletion option). Those genes or protein sequences with more than 50 substitutions per site were considered unrelated bacteriocins.

Thus, phylogenetic analysis both at genetic and protein levels revealed six bacteriocin groups that we refer to as families (Figure 1): BS, including the bacteriocins Bsa, Hyicin 3682 and BacCH91; EP5, formed by Epidicin 280/Homicorcin and Pep5; NUK, which comprises all the nukacins (Nukacin IVK45, Nukacin KQU-131, Nukacin 3299, and Nukacin ISK-1); GEST, including Gallidermin, Staphylococcin T (StT), and Epidermin; the bacteriocin family named EPI, formed by Epilancin 15X and Epilancin K7, and finally, the family CAPSP, including Capidermicin and BacSp222 bacteriocins. The gene cluster encoding the Aureocin A70 (*aurA*, *B*, *C*, *D*) was not considered as a family because it only codifies for a unique bacteriocin and showed high similarity only comparing the amino acid sequence.

Notably, our arrangement corresponds to a majority of those recently reported [19] but provides more details of the similarities of staphylococcins included in each of the 6 classes proposed by this work. Concretely, within Class I Lantibiotics, our families revealed higher similarities between nukacins (NUK family), Gallidermin, Staphylococcin T (StT), and Epidermin (GEST family) and Bsa, Hyicin 3682 and BacCH91 bacteriocins, considered as BS family. As for Class II, we proposed the family CAPSP (conformed by Capidermicin and BacSp222) due to their higher similarities in nucleotide and amino acid sequence. These bacteriocin families have been used in a previous work of our group for designing PCR primers in order to detect possible bacteriocin genes [21].

## 6. Applications of Bacteriocin-Producing *Staphylococcus* Isolates or Their Staphylococcins

Based on the rise of antibiotic-resistant bacteria, a complementary approach in searching for novel drug formulations is demanded. In this respect, purified or partially purified bacteriocins hold great promise and may ultimately be employed as pharmabiotics and/or novel alternatives to existing antibiotics [179]. Moreover, the activity of conventional antimicrobials can be enhanced when combined with novel and often naturally derived antimicrobials [17,180]. It is to note the antimicrobial properties of some staphylococcins alone or in combination with other antimicrobials with high interest to be used in the clinical field, both veterinary and human medicine [7,19,180].

There are few studies concerning the use of staphylococcins carried out in vivo, having a need for in vitro validation before their use in clinical trials both in animal models and in humans. In this respect, the pharmacokinetic parameters of the host [181], the potential bacteriocin-induced toxicity [182], and the route of administration must be considered.

Several studies have tested some staphylococcins in animals. Murine models have been used to analyze the possible role of Staphylococcin 1580 for inhibition of caries [183], Lysostaphin for treating MRSA wounds, pneumonia, and/or systemic infections [184,185,186], or as an alternative for mastitis produced by *S. aureus* [187] and MP1 for skin infections produced by MRSA [188]. Epidermicin NI01 has been tested in the *Galleria mellonella* larvae model [189] and for eradicating nasal colonization of MRSA in rats [190] with very promising results. Human studies have also been performed, and the bacteriocin-producing *S. hominis* ShA9 has been reported as a good alternative to control *S. aureus* during skin dysbiosis and other diseases such as atopic dermatitis [34,191].

Skin infections, especially skin and soft tissue infections (SSTIs) caused by *S. aureus*, are among the most common infections in the world and have been one of the most studied. In the same way, it has been reported that *S. aureus* nasal carriers suffer from infective processes or present a low richness of species in their nasal microbiota, which can precede disease. Moreover, MRSA and specific genetic lineages of *S. aureus* (MRSA-CC398) present emerging antibiotic resistance determinants of special interest. Therefore, being able to control the high prevalence of this multidrug-resistant microorganism in the skin and nasal microbiota of pigs and in-contact humans is a public health challenge.

One new and emerging potential application of staphylococcins or bacteriocin-producing staphylococci is their interesting role in human microbiota modulation [192]. One applied example consists of the balance of skin microbiome in atopic dermatitis, made by CoNS strains to compete or limit *S. aureus* growth, including MRSA [34]. In this respect, several bacteriocin-producing CoNS isolates of skin and mucous tissues with interesting antimicrobial activities commonly against potentially pathogenic Gram-positive and, in a few cases, also in Gram-negative microorganisms, have been reported [49,51,193]. Due to the great potential of bacteriocins, especially those produced by commensal isolates, the identification and characterization of novel antimicrobial peptides should be a clear goal [34,188,191].

On the other hand, bovine mastitis is one of the most persistent and economically significant diseases affecting dairy cattle worldwide. *S. aureus* and *Streptococcus* spp. are the most common etiologic agents involved in bovine mastitis [194,195]. In recent years, the emergence of resistance and the increasingly strict regulations on dairy farms regarding the use of these drugs in animal production has forced the development of alternatives, such as bacteriocins, for the control and prevention of this disease [196]. As mentioned before, Lysostaphin has been tested in a murine mastitis model [187]. Recently, ex vivo and in vitro assays have been carried out with Lysostaphin and with other staphylococcins such as Aureocin A53 and Aureocin A70 [19].

Apart from the antimicrobial activity of bacteriocins produced by *Staphylococcus* described over time, anti-virus, anti-inflammatory, and immunomodulation activities have also been recently reported [133,197,198,199,200,201,202,203,204]. In this respect, one of the most intriguing new fields of investigation is the study of bacteriocins as potential anti-cancer and anti-tubercular agents [205,206,207]. Moreover, recent studies have shown the potential use of staphylococcins or *Staphylococcus*-producing isolates as bio preservatives in meat to assure the microbial shelf-life of the product [208] or as anti-fungic agents to prevent toxigenic molds [209].

However, it is important not only to discover new bacteriocins and antagonism activities but also to test for toxicity to prove their safe use in a preclinical phase (in vivo antimicrobial and/or toxicity effects) as candidates for therapeutic processes.

To this end, biotechnological techniques such as bioengineering or chemical synthesis of bacteriocins can be important tools to improve the antimicrobial activity of bacteriocins, change their physicochemical properties, or reduce the cost of production.

## 7. Emerging Concerns Associated with the Use of Staphylococcins

Apart from the antimicrobial properties exhibited by bacteriocins that make them suitable antimicrobial agents, various concerns associated with their applications in human and animal medicine, food production, and industries have emerged. Among the emerging concerns associated with staphylococcin application, here we will give an overview of their safety in host cells as their toxicity, immunogenicity, bioavailability and absorption, exposure and development of resistance, and the legal framework.

Staphylococcins, as well as other bacteriocins, are generally considered safe antimicrobials and therapeutic substances. However, some staphylococcins have been reported to be cytotoxic to mammalian cell lines, usually at high concentrations [7]. To a large extent, bacteriocins’ cytotoxicity to eucaryotic cells depends on the concentration, purity, composition, and type of eucaryotic cell line used [210,211]. Therefore, since the cytotoxicity of bacteriocins is often evaluated using different concentrations and types of eucaryotic cell lines, it is difficult to generalize and/or compare the cytotoxicity levels. For a comparative safety evaluation, it is necessary to have a consensus on the type of assay, the concentration of staphylococcin, the composition, and the type of cell line to be used.

Regarding the safety and immunogenicity of staphylococcins, it has been reported that the use of bioactive molecules with undesirable immune responses could be detrimental to the host [212,213,214]. Therefore, the assessment of bacteriocins’ immunogenicity profile should be considered crucial and necessary, especially when they are intended for use in the food industry or as biotherapeutics in humans or animals.

Another concern associated with the use of staphylococcins in the food industry or as a therapeutic strategy in humans or animals is the potential risk for the development of resistance upon prolonged exposure to the target or spoilage of pathogenic microorganisms. The resistance development to bacteriocins has been reported to be either (1) intrinsically (innate) within specific genera or related strains or (2) acquired, i.e., resistance developed from a previously susceptible strain [215,216]. However, our understanding of the potential for bacteriocin resistance development has been revealed primarily from experiments performed under laboratory conditions [217]. In this respect, the bacteriocins most studied for the development of resistance are nisin, lacticin 3147, and pediocin-like bacteriocins [216], and regarding staphylococccin resistance, lysostaphin is the most studied so far [218].

Finally, there is a lack of a universal consensus on the legal and regulatory aspects of the use of bacteriocins. Although several bacteriocin-producing microorganisms have attained the ‘generally regarded as safe (GRAS)’ status, it is generally necessary to achieve the guidelines for the approval of bacteriocins either as food additives/preservatives, technological or therapeutic agents depending on their intended use and the subsisting laws of the particular country.

## 8. Conclusions

Bacteriocin-producing staphylococci, especially the commensal CoNS of human and animal microbiota, provide an excellent model to find bacteriocins that could be promising candidates to combat AMR and to compete against pathogens or protect against infections. Moreover, staphylococcins have steadily shown great potential and are being considered for potential applications in clinical, veterinary, food, and biotechnology.

However, characterizing and mining staphylococci for staphylococcins could be demanding. Therefore, this review provides comprehensive and up-to-date approaches for the search, characterization, and evaluation of staphylococcins from staphylococci of different origins. For the first time, we developed a universal nucleotide and amino acid-based phylogeny of all the fully characterized and known staphylococcins. We believe that these resources will undoubtedly help and spur researchers’ interest in exploring staphylococci and advancing the science and application of staphylococcins.

## Figures and Tables

**Table 3:** Description of the staphylococcal bacteriocin sequences used in this study ^a^.

Bacteriocin	Gene ^a^	GenBank Accession No.	GenePept Accession No. ^a^	UniProt	Gene Size (bp)	Protein Size (Da)
Staphylococcin C55	*sacaA*	AF147744	AAD47011	Q9S4D3	188	3339
	*sacbA*		AAD47012	Q9S4D2	203	2993
Aureocin A53	*aucA*	AF447813	AAN71834	Q8GPI4	142	6012.5
Aureocin A70/ Aureocin 4181	*aurD*	AF241888*/MK796167	AAK73555		95	3147.7 ± 1.5
	*aurC*		AAK73554		95	2983.6 ± 1.5
	*aurB*		AAK73553		92	2824.4 ± 1.5
	***aurA***		**AAK73552**		95	2951.5 ± 1.5
BacCH91	*bacCH91*	JQ655767	AFN42846	I6XG59	144	2074.9
Bsa	***bsaA2***		**BAB95630**	A0A0H3K3P8	143	2089
	*bsaA1*	BA000033	BAB95631	A0A0H3JXA5	143	2281
Aureocyclin 4185	*acIA*	KF836421	ATV90647		195	
BacSp222	*bacSp222*	CP011490	ALI97662	A0A0P0C3P7	150	5921.92
Hyicin/Agneticin 3682	*hyiA*	KY021154	ARD24445	A0A1V0JZL0	144	2139
Hyicin 4244	*hycS*	KY887472	ASL69762	A0A221C8V1	128	3274
Capidermicin	*orf4*	MN234131	QFR37570	A0A5P8N9U9	153	5438
Endopeptidase ALE-1	*ale-1*	D86328	BAA13069	O05156	1089	39,350
NisinJ	*nsj*	NZ_MN602039	QGN18867		183	
Gallidermin/Staphylococcin T (StT)	*gdmA*	U61158 ^b^	AAB61135 ^b^	P21838	159	2165.6
Epidermin	*epiA*	X62386	P08136	P08136	156	2151
Epidermicin NI01	*ecdA*	JQ025382	AFD03077	H9BG66	390	6074
Epicidin 280/Homicorcin	*eciA*	Y14023	CAA74348	O54220	90	3133 ± 1.5 and 3136 ± 1.5
Pep5	*pepA*	Z49865	CAA90023	P19578	183	6575.4 ± 1.7
Epilancin 15X	*elxA*	JQ979180	P86047	P86047	168	3173
Epilancin K7	*elkA*	U20348	AAA79236	Q57312	165	3032 ± 1.5
NukacinIVK45	*nukA*	KP702950	AKQ51579		173	2940
Nukacin KQU-131	*nkqA*	AB432987	BAG70955	B5MFD0	173	3003.97
Nukacin 3299	*nukA*	GQ380548	ACU82391	E0WX65	173	2957.3
Nukacin ISK-1	*nukA*	AB125341	BAD01007	Q9KWM4	173	783 (g/mol)
Lugdunin	*lugA*	CP020406	ARB77241		7124	
	*lugC*		ARB77243		8813	
	***lugD***		**ARB77244**		1739	
Lysostaphin	*lss*	U66883	P10547	P10547	1482	
Hominicin	- ^c^	- ^c^	WP_152903494		- ^c^	2038.4
Micrococcin P1 ^d^	*tclE*	KM613043.1	AIU53942.1	Q9F9L4	150	1144.4

^a^ In the cases in which several genes for a bacteriocin have been identified, the coding genes are marked in bold. ^b^ Bacteriocins with high similarities between their coding gene sequence as the case of Gallidermin and Staphylococcin T (StT) or Epicidin 280 and Homicorcin. ^c^ The nucleotide sequence for Hominicin is not available. ^d^ The reference accession number is the one of *Macrococcus caseolyticus.*

## Data Availability

Not applicable.
